# Fasting serum fructose is associated with risk of gestational diabetes mellitus

**DOI:** 10.1186/s12884-022-04768-y

**Published:** 2022-05-28

**Authors:** Hongmei Zhang, Xiaoyong Li, Yixin Niu, Zhen Yang, Youli Lu, Qing Su, Li Qin

**Affiliations:** 1 Department of Endocrinology, Xinhua Hospital, School of Medicine, Shanghai Jiaotong University, Shanghai, China; 2 Shanghai Xuhui Central Hospital/Zhongshan-Xuhui Hospital, Fudan University, Shanghai, China; 3 Shanghai Clinical Center, Chinese Academy of Science, Shanghai, China; 4 Department of Endocrinology, Xinhua Hospital, Chongming Branch, School of Medicine, Shanghai Jiaotong University, Shanghai, China

**Keywords:** Fasting, Fructose, Incidence, Gestational diabetes mellitus

## Abstract

**Objective:**

To investigate the association of fasting serum fructose concentrations and the incidence of GDM.

**Research design and methods:**

Five hundred twenty six pregnant women who attended the obstetric clinic of Xinhua Hospital, Chongming Branch were recruited prospectively from September 2019 to November 2020. Fasting serum fructose concentrations were measured by a validated liquid chromatography–tandem mass spectrometry method. GDM was diagnosed according to the criteria of the IADPSG. Independent sample t-test was used to compare the differences between groups. Multiple stepwise regression analysis was used to estimate the associations of serum fructose and other variables. Multivariate logistic regression models were adopted to evaluate the odds ratios (ORs) for GDM.

**Results:**

Of the 526 pregnant women, 110 were diagnosed with GDM. Fasting fructose concentrations were increased significantly in GDM patients compared to those without GDM (1.30 ug/ml vs 1.16 ug/ml, *p*<0.001). Fasting fructose concentration was independently associated with GDM after adjusting the potential confounders, 1 ug/ml increase in fasting serum fructose level was associated with an 81.1% increased risk of GDM (1.811, [1.155-2.840]). Taking fructose <1.036 ug/ml as the reference, the OR for GDM was significantly higher in fructose ≥1.036 ug/ml group (OR, 1.669; 95% CI, 1.031–2.701) after all the potential confounders were adjusted.

**Conclusions:**

Increased fasting serum fructose levels were independently associated with the incidence of GDM.

## Introduction

Gestational diabetes mellitus (GDM), the most common metabolic complication of pregnancy, is defined as abnormal glucose tolerance with onset or first recognition in the second and third trimester of pregnancy [1]. GDM carries a potentially important risk for severe pregnancy complications for both mother and child [2]. Women with GDM have a considerably increased risk of developing type 2 diabetes mellitus (T2DM) after pregnancy [3], while their offspring have a longer-term risk of obesity and glucose intolerance in life [4]. Along with the increasing obesity prevalence and older maternal age, the incidence of GDM is growing worldwide, causing a major economic burden on public health [5]. The mechanism of the development of GDM is not fully understood. Traditional risk factors include obesity, ethnicity, older maternal age, glycosuria, and previous adverse pregnancy outcomes [6], but these risk factors have limited predictive value, leading to some women with GDM not getting appropriate treatment [7]. Lately, there is research reported that combination of risk factors like BMI, abdominal circumference, fasting glycemia, etc., could determine a high risk of GDM [8], and common genetic risk variants were associated with GDM risk [9]. Finding risk factors of GDM is very important, early detection and proper treatment of GDM could improve health outcomes [10].

Fructose, the isomer of glucose, is a six-carbon monosaccharide which is one of the main components of sugar-sweetened beverages (SSBs) and fruit juice. Dietary fructose is the major source of exogenous fructose. In addition to the majority source of exogenous, animals and humans can also produce fructose endogenously [11]. The endogenous fructose is synthesized through activation of the aldose reductase and sorbitol dehydrogenase. This is the only known pathway of fructose generating in humans and most mammals. At micromolar levels, blood fructose is very reactive in vivo and plays a critical role in mediating the pathology processes. It has been reported that endogenous fructose production and metabolism are involved in the pathogenesis of metabolic syndrome [12]. Higher fasting serum fructose levels were independent risk factors for increased incident type 2 diabetes [13]. So far, the association of fasting serum fructose with GDM has not been fully investigated. In the present study, we are going to explore the relationship between fasting serum fructose concentrations and the GDM in a population of pregnant women without diabetes history.

## Methods

### Study subjects

The study subjects in this study were from the obstetric clinic of Xinhua Hospital, Chongming Branch, School of Medicine, Shanghai Jiaotong University. We collected the information of 526 pregnant women prospectively from September 2019 to November 2020. All of the participants signed the informed consent. The study was approved by the Institutional Review Board of Xinhua Hospital affiliated to Shanghai Jiaotong University School of Medicine. In order to exclude the influence of age on hepatic endogenous fasting fructose production, we only recruited pregnant women aged 25-35. Subjects with a previous diabetes history or a family history of diabetes were all excluded. Subjects taking the drug that affects blood glucose levels were also excluded.

### Measurement of clinical variables

At the time of 24-28 weeks of pregnancy, after fasting for more than 10 hours, all participants underwent a 75g glucose Oral Glucose Tolerance Test (OGTT). The fasting blood, OGTT 1h, and OGTT 2h blood were collected for testing blood glucose to diagnose GDM. At the same time, serum triglycerides (TG), total cholesterol (TC), high-density lipoprotein cholesterol (HDL-C), low-density lipoprotein cholesterol (LDL-C), serum alanine aminotransferase (ALT), γ-glutamyltransferase (GGT), serum creatinine (Scr), and uric acid (UA) were measured using an automatic analyzer (Hitachi 7080; Tokyo, Japan). Serum insulin was tested using RIA (Linco Research, St.Charles, MO, USA). HbA1c was measured using high-performance liquid chromatography (HPLC). Anthropometric measurements were conducted by 2 well-trained nurses. Bodyweight before pregnancy and SSBs consumption were reported by the participants themselves. The frequency of SSBs consumption ranged from times per day to times per week. The usual quantity of SSB consumed was estimated as a standard bottle (250 mL). A typical serving of SSB was defined as 12 oz (360 mL).

### Measurement of blood fructose concentration

When the participants underwent the OGTT at 24-28 weeks of gestation, fasting blood was saved to test fasting fructose. Fasting serum fructose concentrations were measured by a validated liquid chromatography–tandem mass spectrometry method in duplicate as described in a previous article [13]. Briefly, 50 μL serum samples were mixed with 10 μL internal standard (D-fructose-^13^C_6_) and 150 μL methanol, vortexed for 10 s, and then centrifuged. Afterwards, 50 μL of the supernatant was transferred into a tube and mixed with 100 mL 75% aqueous methanol containing 1-ethyl-3- (3-dimethylaminopropyl) carbodiimide hydrochloride (200 mmol/L, Sigma-Aldrich) and 3-nitropheylhydrazine hydrochloride (150 mmol/L, Sigma-Aldrich). The mixture was reacted at 50°C for 60 minutes. After reaction, 350 μL of water was added to the mixture, then the solution was stored at -15°C for assay.

The solution was analyzed by negative ion ultra-performance liquid chromatography–electrospray ionization–mass spectrometry in a multiple reaction monitor mode performed on an UltiMate 3000 RSLC System (Thermo Fisher Scientific, Sunnyvale, CA) coupled to an API 4000 mass spectrometer (SCIEX, Concord, ON, Canada). Chromatographic separations were performed on a Waters ACQUITY UPLC HSS PFP (2.1×150 mm, 1.7 μm) column using water: formic acid (100:0.1 v/v; solvent A) and acetonitrile: formic acid (100:0.1 v/v; solvent B) as the mobile phase for gradient elution at a flow rate of 0.2 mL/min. The mass transitions were charge/mass ratio 314.1→236.0 for fructose and 320.1→240.0 for D-fructose-^13^C_6_, respectively. All the data were acquired and processed using Analyst 1.6 software (SCIEX). Coefficient of variation of the assay was assessed by repeatedly analyzing quality control samples. Inter-assay coefficient of variation was 2.1% for fructose measurements.

### Diagnosis of GDM

Depending on guidelines, GDM can be diagnosed either at any time during pregnancy, or in 24-28 weeks [14]. In our study, the enrolled pregnant women were all with normal fasting blood glucose (FBG) levels in the first trimester, so GDM was screened at 24-28 weeks of pregnancy. GDM was diagnosed according to the criteria of the International Association Diabetes Pregnancy Study Groups (IADPSG), fasting blood glucose≥5.1mmol/L, or 1-h OGTT value ≥10mmol/L, or 2-h OGTT value ≥8.5mmol/L was diagnosed with GDM.

### Statistical analysis

For statistical analysis, we used SPSS 22 software (SPSS Inc., Chicago, IL). Data were demonstrated as means ± SD, median (interquartile range). Independent sample t-test was used to compare the differences between groups. Multiple stepwise regression analysis was used to estimate the associations of serum fructose and other variables. Multivariate logistic regression models were adopted to evaluate the odds ratios (ORs) for GDM. Potential confounding variables including age, blood pressure, BMI before pregnancy, lipid profiles, GGT, and Scr were adjusted in the regression models. *P* values < 0.05 were considered statistically significant. The receiver operating characteristic curve (ROC curve) was used to estimate the cutoff point of fasting fructose for GDM.

## Results

### Clinical and laboratory characteristics of participants with and without GDM

The clinical and laboratory characteristics of the participants in different groups were shown in Table 1. Blood glucose levels, SBP, BMI before pregnancy, HOMA-IR, serum insulin, HDL-C, LDL-C, TG, GGT, Scr, and UA levels were increased significantly in participants with GDM.

**Table 1:** Characteristics of GDM and normal controls

Characteristics	GDM	Normal	*P* value
N	110	416	
Fructose (ug/ml)	1.30±0.51	1.16±0.35	0.001
Age (yr)	29.72±3.08	29.18±2.92	0.104
SBP (mm Hg)	116.49±12.64	113.79±10.42	0.033
DBP (mm Hg)	65.61±26.71	61.22±28.67	0.148
BMI before pregnancy (kg/m^2^)	23.04±4.24	21.79±3.23	0.002
FBG (mmol/l)	4.87±0.56	4.37±0.31	<0.001
OGTT 1h BG (mmol/l)	9.64±1.63	7.19±1.33	<0.001
OGTT 2h BG (mmol/l)	8.47±1.55	6.44±1.00	<0.001
HbA1C (%)	5.19±0.85	4.95±1.99	0.236
Insulin (pmol/l)	55.90 (33.70, 77.70)	37.59 (23.85, 57.95)	<0.001
1h-Insulin (pmol/l)	371.75 (282.65, 555.35)	317.20 (209.00, 458.50)	<0.001
2h-Insulin (pmol/l)	405.90 (263.05, 575.70)	305.65 (197.55, 442.45)	<0.001
HOMA-IR	2.07±1.57	1.21±0.78	<0.001
HDL (mmol/L)	2.65±0.56	2.80±0.58	0.031
LDL (mmol/L)	2.96±0.70	2.78±0.72	0.028
TC (mmol/L)	5.47±0.96	5.31±0.95	0.133
TG (mmol/L)	2.02±1.06	1.62±0.65	<0.001
ALT (U/L)	10 (7, 16)	8 (6, 15)	0.500
GGT (U/L)	14 (10.5, 21.5)	11 (9, 15)	0.001
Scr (umol/L)	39.64±11.10	37.33±7.25	0.016
UA (mg/dL)	0.26±0.11	0.20±0.12	0.029
SSBs (servings/week)	0.31±0.28	0.36±0.25	0.320

Data are presented as means ± SD or median (interquartile range)

*SBP* Systolic blood pressure, *DBP* Diastolic blood pressure, *FBG* Fasting blood glucose, *HDL* High-density lipoprotein cholesterol, *LDL* Low-density lipoprotein cholesterol, *TC* Total cholesterol, *TG* Triglycerides, *ALT* Alanine aminotransferase, *GGT* γ-glutamyltransferase, *Scr* Serum creatinine, *UA* Uric acid

### Blood fructose and GDM

Of the 526 pregnant women, fasting serum fructose concentrations were approximately normally distributed with a mean value of 1.19±0.43 ug/ml. 110 were diagnosed with GDM according to the criteria of the IADPSG. Fasting fructose concentrations were increased significantly in GDM patients compared to those without GDM (1.30±0.51 ug/ml vs 1.16±0.35 ug/ml, *p*<0.001). Fasting fructose concentration was independently associated with GDM after adjusting all the potential confounders like BMI before pregnancy, serum insulin, SSBs consumption, and other related variables. The ORs and 95%CIs for GDM were 1.811 (1.155-2.840), 1 ug/ml increase in fasting serum fructose level was associated with an 81.1% increased risk of GDM (Table 2).

**Table 2:** Logistic regression analysis showing variables independently associated with GDM

Independent variables	β	Exp (β) 95% CI	*P* value
Fructose	0.594	1.811 (1.155-2.840)	<0.01
BMI before pregnancy	0.094	1.098 (1.034-1.166)	<0.001
HOMA-IR	0.728	2.070 (1.652-2.594)	<0.001
GGT	0.026	1.026 (1.008-1.045)	<0.01

The variables entered in the analysis also included age, SBP, DBP, TC, HDL, LDL, ALT, Scr, SSBs consumption which were all excluded from the model

In order to find the cutoff value of fructose for GDM, we used the ROC curve and found that fasting fructose of 1.036 ug/ml was the proper cutoff value for GDM (area under the curve was 0.631, sensitivity and specificity were 60.7% and 65.9% respectively). Table 3 demonstrated the ORs and 95%CIs for GDM by different fructose categories. Taking fructose <1.036 ug/ml as the reference, the OR for GDM was significantly higher in fructose ≥1.036 ug/ml group both in the crude model (OR, 1.841; 95% CI, 1.168–2.902) and in the adjusted model (OR, 1.669; 95% CI, 1.031–2.701).

**Table 3:** Adjusted ORs and 95% CIs for GDM according to fructose categories

Fructose concentration (ug/ml)	n/total	Crude OR (95%CI)	Adjusted OR (95%CI)	
			Model 1	Model 2
<1.036	32/211	1	1	1
≥1.036	78/315	1.841** (1.168-2.902)	1.690* (1.055-2.706)	1.669* (1.031-2.701)

Model 1 adjusted for age and BMI before pregnancy

Model 2 further adjusted for SBP, DBP, TG, TC LDL, HDL, ALT, Scr and HOMA-IR. *p<0.01, ** *p*<0.001

### Variables independently associated with fructose

Multiple stepwise regression analyses showing variables independently associated with fructose were FBG, OGTT 1h blood glucose, OGTT 1h blood glucose, and SBP after adjusting all the possible confounders such as TG, Insulin, ALT, GGT, and Scr (Table 4).

**Table 4:** Multiple stepwise regression analysis showing variables independently associated with fructose

Independent variables	Standardized β	t	*P* value
FBG	0.204	4.310	<0.001
OGTT 1h BG	0.096	2.219	0.027
OGTT 2h BG	0.150	3.474	0.001
SBP	0.150	3.187	<0.001

The analysis also included age, BMI before pregnancy, TG, Insulin, ALT, GGT, and Scr which were all excluded from the model

## Discussion

Fructose, an important dietary source of carbohydrates, is a common monosaccharide that exists naturally in its free form in honey, fruits, and other plants and in a combined form as half of the disaccharide sucrose. Epidemiological studies have demonstrated that sugar consumption, especially in the form of SSBs, contributes to the increased risk of obesity, fatty liver, type 2 diabetes, cardiovascular disease, and mortality [15, 16]. High fructose consumption induces insulin resistance in both experimental animals and humans. Fructose-sweetened beverage consumption for ten constant weeks could cause insulin resistance and glucose intolerance in overweight or obese adults [17]. Fructose has been reported to be related to obesity and metabolic syndrome. Experimental studies have demonstrated that fructose is able to induce leptin resistance and bring about metabolic syndrome in rats [18, 19].

In addition to dietary sources, fructose can be synthesized endogenously in the body by aldose reductase and the polyol pathway. In humans, the mechanism for endogenously producing fructose is from sorbitol as part of the polyol pathway [20]. It is noteworthy that overproduction of hepatic endogenous fructose could lead to systemic metabolic changes [12]. The endogenous fructose could contribute greatly to the pathogenesis of metabolic syndrome. SSB challenge test in a study has shown that circulating fructose levels were increased by 20-fold at 0.5–1 h and returned to baseline at 6 h [13]. Fasting circulating fructose concentration is relatively stable. Fasting blood fructose concentrations were not influenced by high or low fructose diet intake [21]. The association of fasting serum fructose levels with incident diabetes is not affected by SSB consumption, fasting serum fructose concentration could serve as a potential biomarker or contributor to incident diabetes [13].

GDM is a growing health concern. The prevalence of GDM is increasing worldwide along with the diagnostic criteria of IADPSG being applied and advanced maternal age. Because of short-term and long-term adverse health outcomes for both mother and child, understanding risk factors associated with the development of GDM is very significant. There are inadequate data studying associations of fructose with GDM. Animal research has indicated that overload of fructose will lead to an elevated incidence of GDM as well as altered maternal, fetal and offspring metabolic function [22]. However, the animals in these studies were receiving high doses of fructose exogenously. As for the association of endogenously produced fructose with GDM, it has not been reported so far.

Since fasting serum fructose concentrations were associated with incident diabetes in adults on ordinary diet [13], it is worthwhile investigating the association between fasting serum fructose concentrations and the incidence of GDM. In the current study, we discovered that increased fasting serum fructose levels were significantly associated with the incidence of GDM. This association was independent of other established risk factors. To our knowledge, this is the first study investigating the association between fasting serum fructose concentration and the incidence of GDM.

Serum fructose concentrations were associated with hyperglycemia [23]. Fructose and glucose are structural isomers, fructose can be enzymatically synthesized through the polyol pathway which is a metabolic route constituted by two enzymes, aldose reductase and sorbitol dehydrogenase [20]. The polyol pathway may play an important role in the increment of serum fructose levels. This pathway is reported to be responsible for the increased fructose concentrations in many tissues of patients with diabetes [24]. Some research has shown that hyperglycemia can stimulate aldose reductase expression [20]. Vise versa, increased fructose levels may cause increased blood glucose, but the mechanism is still not very clear. Our results showed that endogenous fructose is a possible risk factor for GDM, and elevated fasting fructose is related to GDM. In a previous study, the average fasting fructose values were 0.85 ug/ml in those who developed type 2 diabetes while the mean fasting blood glucose levels were 5.83 mmol/L [13], in this study, the fructose values were much higher in GDM (1.3 ug/ml). Since the average blood glucose levels in GDM are lower than that in the general population reported before [13], the higher value of fructose in GDM is not due to higher glucose levels alone, there may be other effects of pregnancy on endogenous fructose levels. The placenta has been reported to synthesize sorbitol and this may raise fructose concentrations [25]. Impaired fructose metabolism in the liver and disrupted transport system for fructose might also be involved in elevated fasting fructose levels in GDM. More research is needed to verify these assumptions.

Although circulating in micromole concentrations, fructose is much more reactive and may be comparable to glucose in respect of mediating pathology [26, 27]. In GDM, whether elevated fructose levels are related to adverse pregnancy outcomes in women and their offspring are not fully understood, further research is needed in this aspect.

Research shows that fructose promotes hyperlipidemia and hyperuricemia. However, most of these studies used either very-high-fructose diets or fructose intravenously in high doses, the studied humans or animals were not on ordinary diets [28–30]. In this study, we found no significant correlation between fasting serum fructose concentration and TG or uric acid. But we found SBP was independently related to blood fructose in fasting state.

Our research also has limitations. Firstly, it is not a prospective study, we can’t confirm a causal link between fasting fructose concentration and GDM. Secondly, we didn’t have data on pregnancy outcomes in women and their offspring, the relationship between increased fructose concentrations and pregnancy outcomes couldn’t be confirmed, more research is needed in the future. Thirdly, this is a single center study which may affect the universality of the results.

In summary, we found that increased fasting serum fructose levels were significantly associated with the incidence of GDM. This association was independent of other established risk factors. Fasting blood fructose might serve as a risk factor for gestational diabetes mellitus.

## Data Availability

The datasets used and analyzed during the current study are available from the corresponding author on request.

## References

[1] Chiefari E, Arcidiacono B, Foti D, Brunetti A (2017). Gestational diabetes mellitus: an updated overview. J Endocrinol Invest.

[2] Metzger BE, Lowe LP, Dyer AR, Trimble ER, Chaovarindr U, Coustan DR, Hadden DR, McCance DR, Hod M, Group HSCR (2008). Hyperglycemia and adverse pregnancy outcomes. N Engl J Med.

[3] Bellamy L, Casas JP, Hingorani AD, Williams D (2009). Type 2 diabetes mellitus after gestational diabetes: a systematic review and meta-analysis. Lancet.

[4] Group HSCR (2002). The Hyperglycemia and Adverse Pregnancy Outcome (HAPO) Study. Int J Gynaecol Obstet.

[5] Zhu Y, Zhang C (2016). Prevalence of Gestational Diabetes and Risk of Progression to Type 2 Diabetes: a Global Perspective. Curr Diab Rep.

[6] Zhang C, Rawal S, Chong YS (2016). Risk factors for gestational diabetes: is prevention possible?. Diabetologia.

[7] Miailhe G, Kayem G, Girard G, Legardeur H, Mandelbrot L (2015). Selective rather than universal screening for gestational diabetes mellitus?. Eur J Obstet Gynecol Reprod Biol.

[8] Popova PV, Grineva EN, Gerasimov AS, Kravchuk EN, Ryazantseva EM, Shelepova ES (2015). The new combination of risk factors determining a high risk of gestational diabetes mellitus. Minerva Endocrinol.

[9] Popova PV, Klyushina AA, Vasilyeva LB, Tkachuk AS, Vasukova EA, Anopova AD, Pustozerov EA, Gorelova IV, Kravchuk EN, Li O (2021). Association of Common Genetic Risk Variants With Gestational Diabetes Mellitus and Their Role in GDM Prediction. Front Endocrinol (Lausanne).

[10] Duran A, Saenz S, Torrejon MJ, Bordiu E, Del Valle L, Galindo M, Perez N, Herraiz MA, Izquierdo N, Rubio MA (2014). Introduction of IADPSG criteria for the screening and diagnosis of gestational diabetes mellitus results in improved pregnancy outcomes at a lower cost in a large cohort of pregnant women: the St. Carlos Gestational Diabetes Study. Diabetes Care.

[11] Barone S, Fussell SL, Singh AK, Lucas F, Xu J, Kim C, Wu X, Yu Y, Amlal H, Seidler U (2009). Slc2a5 (Glut5) is essential for the absorption of fructose in the intestine and generation of fructose-induced hypertension. J Biol Chem.

[12] Lanaspa MA, Ishimoto T, Li N, Cicerchi C, Orlicky DJ, Ruzycki P, Rivard C, Inaba S, Roncal-Jimenez CA, Bales ES (2013). Endogenous fructose production and metabolism in the liver contributes to the development of metabolic syndrome. Nat Commun.

[13] Chen Y, Lin H, Qin L, Lu Y, Zhao L, Xia M, Jiang J, Li X, Yu C, Zong G (2020). Fasting Serum Fructose Levels Are Associated With Risk of Incident Type 2 Diabetes in Middle-Aged and Older Chinese Population. Diabetes Care.

[14] Popova P, Castorino K, Grineva EN, Kerr D. Gestational diabetes mellitus diagnosis and treatment goals: measurement and measures. Minerva Endocrinol. 2016;41(4):421-32. 26824326

[15] Imamura F, O’Connor L, Ye Z, Mursu J, Hayashino Y, Bhupathiraju SN, Forouhi NG (2015). Consumption of sugar sweetened beverages, artificially sweetened beverages, and fruit juice and incidence of type 2 diabetes: systematic review, meta-analysis, and estimation of population attributable fraction. BMJ.

[16] Choo VL, Viguiliouk E, Blanco Mejia S, Cozma AI, Khan TA, Ha V, Wolever TMS, Leiter LA, Vuksan V, Kendall CWC (2018). Food sources of fructose-containing sugars and glycaemic control: systematic review and meta-analysis of controlled intervention studies. BMJ.

[17] Stanhope KL, Schwarz JM, Keim NL, Griffen SC, Bremer AA, Graham JL, Hatcher B, Cox CL, Dyachenko A, Zhang W (2009). Consuming fructose-sweetened, not glucose-sweetened, beverages increases visceral adiposity and lipids and decreases insulin sensitivity in overweight/obese humans. J Clin Invest.

[18] Tappy L, Le KA (2010). Metabolic effects of fructose and the worldwide increase in obesity. Physiol Rev.

[19] Johnson RJ, Perez-Pozo SE, Sautin YY, Manitius J, Sanchez-Lozada LG, Feig DI, Shafiu M, Segal M, Glassock RJ, Shimada M (2009). Hypothesis: could excessive fructose intake and uric acid cause type 2 diabetes?. Endocr Rev.

[20] Andres-Hernando A, Johnson RJ, Lanaspa MA (2019). Endogenous fructose production: what do we know and how relevant is it?. Curr Opin Clin Nutr Metab Care.

[21] Patel C, Sugimoto K, Douard V, Shah A, Inui H, Yamanouchi T, Ferraris RP (2015). Effect of dietary fructose on portal and systemic serum fructose levels in rats and in KHK-/- and GLUT5-/- mice. Am J Physiol Gastrointest Liver Physiol.

[22] Regnault TR, Gentili S, Sarr O, Toop CR, Sloboda DM (2013). Fructose, pregnancy and later life impacts. Clin Exp Pharmacol Physiol.

[23] Kawasaki T, Akanuma H, Yamanouchi T (2002). Increased fructose concentrations in blood and urine in patients with diabetes. Diabetes Care.

[24] Hamada Y, Nakamura J, Naruse K, Komori T, Kato K, Kasuya Y, Nagai R, Horiuchi S, Hotta N (2000). Epalrestat, an aldose reductase ihibitor, reduces the levels of Nepsilon-(carboxymethyl)lysine protein adducts and their precursors in erythrocytes from diabetic patients. Diabetes Care.

[25] Hwang JJ, Johnson A, Cline G, Belfort-DeAguiar R, Snegovskikh D, Khokhar B, Han CS, Sherwin RS (2015). Fructose levels are markedly elevated in cerebrospinal fluid compared to plasma in pregnant women. PLoS One.

[26] McPherson JD, Shilton BH, Walton DJ (1988). Role of fructose in glycation and cross-linking of proteins. Biochemistry.

[27] Hayward LD, Angyal SJ (1977). A Symmetry rule for the Circular Dichroism of reducing sugars, and the proportion of Carbonyl forms in Aqueous solutions thereof. Carbohydr Res.

[28] Lee MK, Miles PD, Khoursheed M, Gao KM, Moossa AR, Olefsky JM (1994). Metabolic effects of troglitazone on fructose-induced insulin resistance in the rat. Diabetes.

[29] Thorburn AW, Storlien LH, Jenkins AB, Khouri S, Kraegen EW (1989). Fructose-induced in vivo insulin resistance and elevated plasma triglyceride levels in rats. Am J Clin Nutr.

[30] Woods HF, Alberti KG (1972). Dangers of intravenous fructose. Lancet.

